# Secondary Tricuspid Regurgitation: Pathophysiology, Incidence and Prognosis

**DOI:** 10.3389/fcvm.2021.701243

**Published:** 2021-07-22

**Authors:** Muhammed Gerçek, Volker Rudolph

**Affiliations:** Clinic for General and Interventional Cardiology/Angiology, Herz- und Diabeteszentrum NRW, Ruhr-Universität Bochum, Bad Oeynhausen, Germany

**Keywords:** secondary tricuspid regurgitation, valvular disease, primary tricuspid regurgitation, isolated tricuspid regurgitation, atrioventricular valve

## Abstract

Tricuspid regurgitation (TR) can be divided into primary and secondary origins. Primary TR is mostly caused by infective endocarditis, leaflet perforation, entrapment after device placement and congenital abnormalities. The natural cause of secondary (functional) TR is not well-understood and underdiagnoses is likely. Because symptoms such as ascites, edema and hepatomegaly usually manifest at a late state, assessment of TR is challenging requiring a multiparametric approach. Secondary TR can be subdivided into four morphologic types according to the underlying mechanism: Left-heart related TR, precapillary pulmonary hypertension related TR, right ventricular disease related TR and isolated TR.

## Introduction

Tricuspid regurgitation (TR) has long been the most neglected valvular disease. This is mainly due to so far limited treatment options. On the one hand, conservative therapy results in resistance to diuretic treatment while surgical therapy on the other hand is associated with high in-hospital mortality (8.8%) (1). With the introduction of transcatheter tricuspid valve treatment options, which have shown promising results, the forgotten valve has finally emerged from the shadows.

This review aims to provide insight into the pathophysiology, incidence and prognosis of secondary tricuspid regurgitation in particular.

## Pathophysiology and Prevalence of Primary Tricuspid Regurgitation

**Figure 1:**
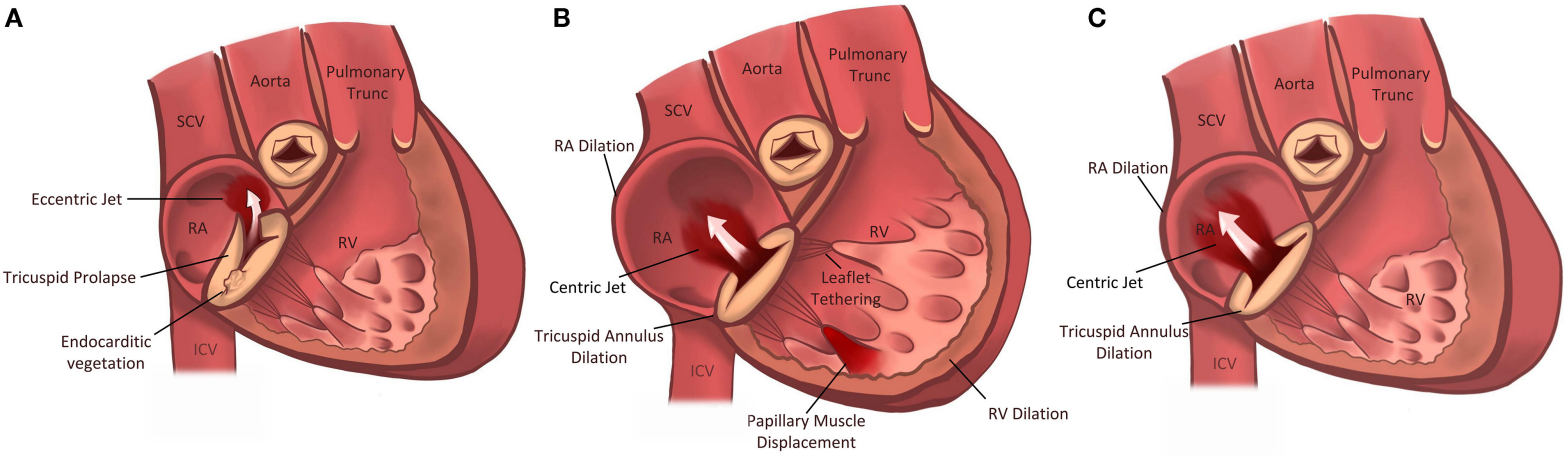
Pathophysiologic Subdivision of Tricuspid Regurgitation. Primary tricuspid regurgitation is caused by abnormalities/damage on the tricuspid valve apparatus **(A)** (e.g., prolapse of the leaflet or endocarditis). Left heart related tricuspid regurgitation and precapillary pulmonary tricuspid regurgitation **(B)** are caused by dilation of the right ventricle, papillary muscle displacement and tethering of the tricuspid valve leaflets with malcoaptation. In isolated tricuspid regurgitation **(C)** the tricuspid annulus is pronouncedly dilated due to dilation of the right atrium in the presence of atrial fibrillation or diastolic dysfunction.

Akin to mitral regurgitation, TR may be of primary (degenerative) or secondary (functional) origin. Primary tricuspid regurgitation (PTR) occurs less frequently (8–10% of all-cause TR) (2). In PTR abnormalities of the tricuspid valve apparatus may be of congenital or acquired origin. Apical displacement of the tricuspid leaflets that arise directly from the right ventricle without being linked to chordae is the most common congenital cause of primary TR (Ebstein's disease) (3). Acquired primary tricuspid regurgitation is mostly caused by leaflet perforation and entrapment following device placement (4). Considering a continuously aging population with an increased need for cardiac pacemaker-implantation, the prevalence of pacemaker/lead-induced TR may increase and should be considered in future device selection as novel techniques such as his bundle pacing and leadless pacemakers are quickly becoming available (5). Another important entity of PTR is endocarditis (Figure 1A), which makes up 17% of all endocarditis cases, predominantly occurs in males and is very often a consequence of intravenous drug abuse or is also related to implantable devices. It affects the anterior leaflet and manifests with large vegetations in the majority of cases (6, 7). Rarer causes of PTR are chordae rupture following right-ventricular biopsies often seen after cardiac transplantation or hepatically metastased neuro-endocrine tumors (Hedinger syndrome), which involve the heart and particularly the tricuspid valve in 60% resulting in fibrotic stiffening of the leaflets (8).

## Pathophysiology and Incidence of Secondary (Functional) Tricuspid Regurgitation

The natural cause of secondary (functional) tricuspid regurgitation (FTR) is not yet well-understood and in general four types of secondary tricuspid regurgitation are described (9) (Table 1; Figure 1):

- Left heart related tricuspid regurgitation (LH-TR).
- Precapillary pulmonary hypertension related tricuspid regurgitation (PH-TR).
- Right ventricular disease related tricuspid regurgitation (RVD-TR).
- Isolated tricuspid regurgitation (ITR).

**Table 1:** Characteristic of the four types of secondary (functional) tricuspid regurgitation.

**Type of FTR**	**RV dilation**	**Tricuspid annulus**	**RA dilation**	**Leaflet tethering**	**Echocardiography or MRI**
LH-TR	+++ (also mid and apical RV)	++	++	+++	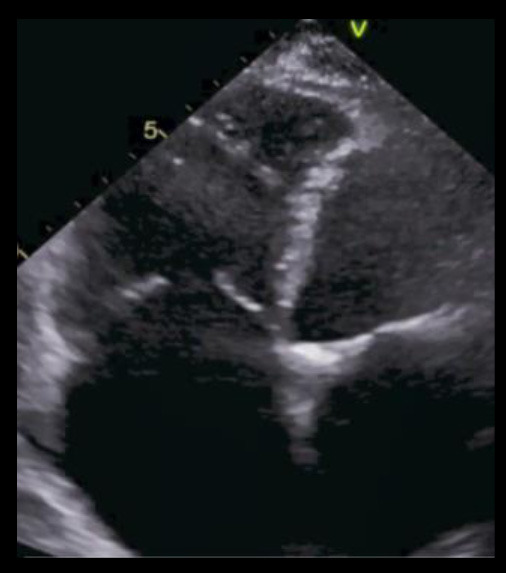
PH-TR	+++ (also mid and apical RV)	++	+++	+++	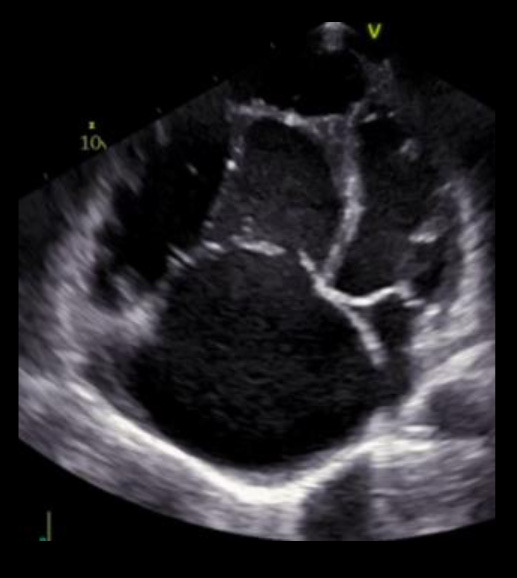
RVD-TR	+++ (Depending on disease etiology)	++	++	+++	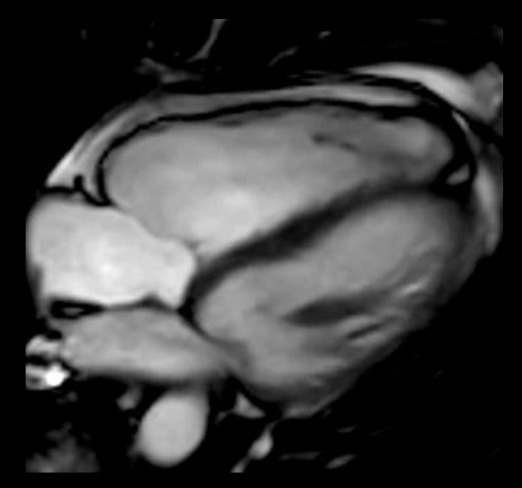
ITR	++ (Particularly at the basal level)	+++	+++	+	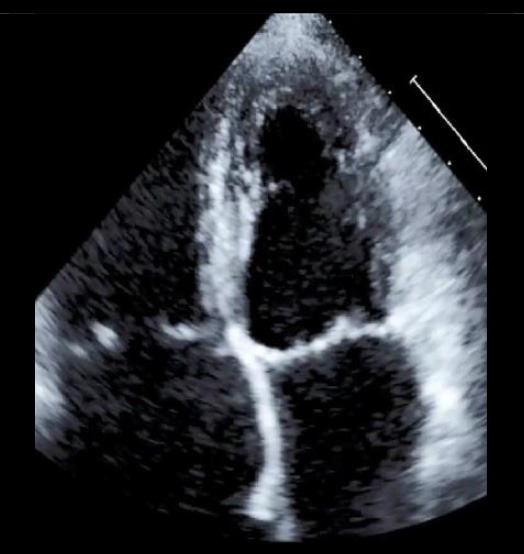

*Term explanation: FTR, functional tricuspid regurgitation; ITR, isolated tricuspid regurgitation; LH-TR, left heart related tricuspid regurgitation; MRI, magnet resonance imaging; PH-TR, precapillary pulmonary hypertension related tricuspid regurgitation; RA, right atrial; RV, right ventricular; RVD, right ventricular disease related tricuspid regurgitation*.

To understand the pathophysiology of FTR it is crucial to understand the anatomy of the right heart and the tricuspid valve. Generally, the tricuspid valve consists of three leaflets but anatomical variants with a variable number of leaflets commonly occur (10). The posterior leaflet is usually smaller than the anterior and septal ones and functional TR often results in an ellipsoid regurgitant orifice along the anteroposterior edge with a shorter septolateral dimension (11). The leafleats are linked via chordae to papillary muscles and therefore tethering following right ventricular remodeling can occur, which in turn may cause TR even in the absence of tricuspid annulus (TA) dilation (Figure 1B) (12).

Functionally however, the tricuspid annulus belongs to the right ventricle (RV) as it is septally fixed, partly consists of a fibrous tissue and is sensitive to pre- and afterload as well as right ventricular and/or atrial dilation (12, 13). Therefore, enlargement of the non-planar and elliptical annulus during tricuspid regurgitation results in a more circular and planar tricuspid architecture (9).

Topilsky et al. described a prevalence of significant TR of 0.55% in 21,020 examined patients. However, 1 in 25 patients older than 75 years presented with a moderate or severe TR whereas all-cause TR was more often diagnosed in women (14).

Characterization of the mechanism of TR is difficult and hampered by the limitations of 2D-echocardiography. Resulting from a lack of standardization and its volume dependence the assessment of TR can be challenging and thus requires a multi-parametric approach including quantitative [e.g., effective regurgitant orifice area (EROA), regurgitant volume], semiquantitative [e.g., annulus dilation, vena cava width, proximal isovelocity surface area (PISA), jet area, hepatic flow, tricuspid valve inflow], and qualitative (e.g., inferior vena cava size, right atrium size, right ventricle size, ventricular septum motion, tricuspid valve morphology, color flow jet, jet contour, flow convergence zone) evaluation in transthoracic and transesophageal echocardiography (15, 16). In this context it is important to mention, that when weighing up treatment options, right ventricular function and geometry is considered an important factor and thus should also be assessed using parameters such as tricuspid annular plane systolic excursion (TAPSE), right ventricular fractional area change (RV-FAC) and right ventricular longitudinal strain (RV-GLS). With the introduction and establishment of 3-D real-time echocardiography the ability to understand and differentiate between the types of TR improved tremendously (11).

However, novel transcatheter therapeutic approaches may require additional cardiac computed tomographic assessments for procedural planning which allow to precisely determine the tricuspid annular size and the neighboring anatomical structures such as the right coronary artery or the coronary sinus thus estimate potential periinterventional risks (17). In some cases, the use of magnetic resonance tomography can be helpful to evaluate right heart function and volume.

## Left-Heart Related Tricuspid Regurgitation

Left-heart related tricuspid regurgitation (LH-TR) is the most common form of FTR caused by left-sided valvular and myocardial disease associated with increased left atrial pressure, pulmonary hypertension and increased RV afterload which leads to RV dilation in particular at the basal level, tricuspid leaflet tethering, tricuspid annulus dilation and leaflet malcoaptation (Figure 1B) (14). Compared to healthy valves the annulus becomes more planar, circular and dilated. Due to RV dilation, TR regurgitation results in a larger RV eccentricity index. Depending on the presence of pulmonary hypertension the RV becomes more elliptical, emphasizing valvular tethering (9, 10).

Topilsky et al. described that in 62.5% of patients who were diagnosed with significant TR left-heart related diseases were the culprit (14). One third of patients with severe mitral regurgitation and one quarter of patients with severe aortic stenosis presented with significant tricuspid regurgitation (at least moderate) (9, 18). Furthermore, heart failure patients with reduced ejection fraction frequently presented with significant tricuspid regurgitation which can progress even despite optimal medical therapy (19, 20). Benfari et al. demonstrated that 88% of 13,026 heart failure patients with reduced ejection fraction showed functional tricuspid regurgitation. Among these 26% were classified as at least moderate (19).

## Precapillary Pulmonary Hypertension Related Tricuspid Regurgitation

Precapillary pulmonary hypertension related tricuspid regurgitation (PH-TR) is usually observed in patients with chronic lung disease, pulmonary thromboembolism, left-to-right shunting and a doppler estimated systolic pulmonary artery pressure of > 50 mmHg (10).

The RV shows a midventricular dilation, a dilation of the tricuspid annulus and the right atrium (Figure 1B). However, leaflet tethering due to lateral and apical papillary muscle displacement seems to be the dominant mechanism in pulmonary hypertension (21, 22).

Functional parameters of the right ventricle such as fractional area change (FAC) and tricuspid annular plane systolic excursion (TAPSE) do not generally show impairment whereas the RV-sphericity index (diameter/length) increases with progression of TR. Right atrial (RA) dilation is also frequently associated with PAH and severe TR (23). Tricuspid valve tenting height and area is also significantly increased. Additionally, along progression of TR leaflet length increases in PAH, whereas tricuspid valve coverage (leaflet length/tenting area) decreases significantly due to RV dilation (24).

Increases in systolic pulmonary artery pressure (PASP) and RV size as well as reduced tricuspid valve coverage are associated with TR progression which leads to progressive right heart failure (24). However, predicting TR progression with baseline RV size, PASP or TA diameter is not yet possible which limits selection of at-risk patients who may benefit from a more aggressive therapeutic approach (24).

In general, when PAH is confirmed, TR is common. Tricuspid regurgitation was present in 96.5% of a PAH population while 60% them were at least of moderate severity (25). The estimated incidence of PAH ranges between 7 and 26 cases per 1 million adults (26).

## Right Ventricular Disease Related Tricuspid Regurgitatio

Intrinsic right ventricular dysfunction in the absence of pulmonary hypertension and diseases such as arrhythmogenic right ventricular cardiomyopathy and inferior cardiac infarction can lead to tricuspid regurgitation due to papillary muscle displacement and malfunction resulting in increased tricuspid leaflet tethering and insufficient leaflet coaptation (27, 28). In arrhythmogenic right ventricular cardiomyopathy in particular, fibro-fatty tissue replacement due to progressive myocyte loss leads to right atrial and ventricular dilation lacking a specific pattern which favors the development of tricuspid regurgitation (27, 29). An incidence value of RVD-TR has not been reported in the literature so far. However, 15% of a cohort of ARVC patients showed a significant TR which contributes to worsening HF by increasing RV filling pressure and decreasing RV forward stroke volume (27).

## Isolated Tricuspid Regurgitation

Isolated (idiopathic) tricuspid regurgitation (ITR) is described as a morphologic type of TR in the absence of left-heart sided causes, pulmonary hypertension or primary right ventricular diseases. Hence, it is recognized as a separate entity (other than secondary TR) (9, 20, 30). In the absence of pronounced pulmonary hypertension, the right ventricle shows a not so dominant elongation but rather a dilation of the basal segments (11). Additionally, 3D-Echocardiography with tricuspid valve analysis using 3D-quantifation software revealed that the tricuspid annulus in patients with ITR was pronouncedly more dilated, planar, circular and dysfunctional than in patients with LH-TR with less leaflet tethering and tenting volume (Figure 1C) (11). Patient characteristics are also different to LH-TR. Isolated TR mostly appears in female patients of advanced age with smaller body surface area, lower likelihood of coronary artery disease and higher rate of arterial hypertension and in particular atrial fibrillation (14). The RA shows a higher degree of enlargement. Rotational and helical blood flow within the RA is hence disrupted, particularly in atrial fibrillation, which may contribute to TR progression (10, 31). Utsonomya et al. described a prevalence of 9.2% of isolated tricuspid regurgitation in patients diagnosed with at least moderate TR (11). Topilsky et al. could show that 8.1% of significant TRs diagnosed in American community residents were of isolated origin (14). Interestingly, diastolic dysfunction (heart failure with preserved ejection fraction; HFpEF) seems to be another key mechanism in isolated TR in patients without atrial fibrillation (20). Interestingly, Mascherbauer et al. found that 51% of routinely followed HFpEF patients had at least moderate secondary TR. Patients with TR had a higher pulmonary vascular resistance, reduced pulmonary compliance, and elevated left ventricular filling pressure compared to those presenting without TR (20). Therefore, tricuspid regurgitation – once diagnosed – should entail further assessment of the left ventricle, in particular with regard to diastolic dysfunction (20) and vice versa patients with diagnosed HFpEF should be monitored for worsening RV function and TR.

## Clinical Outcome and Prognosis of Functional Tricuspid Regurgitation

The prognostic relevance of tricuspid regurgitation has long been recognized. Thus, already in 2004 Nath et al. showed that severe TR was associated with a reduced 1-year mortality of around 64% in over 5,000 patients who were followed over a period of 4 years (2). More recently, the prognostic relevance of TR has been demonstrated for nearly every underlying etiology.

Thus, the presence of at least moderate TR was associated with a significantly increased mortality risk (HR: 2.17; 95% CI: 1.30–3.63) in patients with prior surgical mitral valve replacement (32). Data from the German Mitraclip registry revealed that patients with concomitant severe tricuspid regurgitation who underwent edge-to-edge mitral valve intervention had a higher one-year mortality (HR 2.01; 95% CI 1.25–3.23; *p* = 0.004) as well as MACCE rate (33). Two-year data from the COAPT-Trial also showed that concomitant severe tricuspid regurgitation worsened the clinical outcome of patients (composite rate of death and hospitalization for heart failure 83.0 vs. 64.3%; HR: 1.74; 95% CI: 1.24–2.45; *p* = 0.001) (34). However, interventional mitral valve treatment improved outcome in patients with and without significant tricuspid regurgitation. Using data from the TriValve and TRAMI registries (n_overall_ = 228), Mehr et al. could show that simultaneous mitral and tricuspid interventional therapy in patients with both severe mitral and tricuspid regurgitation was associated with a higher 1-year survival than isolated transcatheter mitral repair (HR 0.52; *p* = 0.02) (35).

Following transcatheter aortic valve replacement for aortic stenosis a large registry study with 34,576 patients revealed that TR severity also correlated with mortality (HR 1.29; 95% CI 1.11–1.50; *p* < 0.001) and readmission (HR 1.27; 95% CI 1.04–1.54; *p* < 0.001) (36). More than mild TR was also found to be associated with increased mortality in patients undergoing surgical aortic replacement (37).

Benfari et al. could highlight that among 13,026 patients increased severity of TR is associated with a lower 5-year survival in heart failure patients with reduced ejection fraction despite optimal medical treatment (HR 1.57 95% CI 1.39–1.78) (19). Bartko et al. emphasized that even moderate TR may be a relevant prognostic factor in patients with HFrEF which is why it should be taken into account when determining the most suitable therapeutic approach (38). With regard to right ventricular diseases as underlying pathology, tricuspid regurgitation has been identified as a prognostic parameter of death or need for heart transplantation during a 10-year follow up of ARVC patients (HR 7.6; 95% CI 2.6–22.0; *p* < 0.001) (27).

Among 727 newly diagnosed PAH patients severe tricuspid regurgitation was shown as an independent predictor for greater 5-year mortality risk (HR 1.83 95 CI 1.38–2.41; *p* < 0.001) (25). Furthermore, among 88 PAH patients TR progression was associated with worsening pulmonary hypertension and poor outcome (HR 3.42; 95% CI 1.73–6.73; *p* < 0.001) (24).

Isolated tricuspid regurgitation is also independently associated with excess mortality and morbidity. Topilsky et al. could demonstrate in a retrospective study with 353 mostly female patients that isolated TR was associated with lower 10-year survival rate (38 ± 7% vs. 70 ± 6%; *p* > 0.001), in particular in presence of atrial fibrillation which may be explained by progressive RA remodeling in atrial fibrillation with higher risk for right heart failure (30). These findings were confirmed by a 15-year survival analysis using data from a large American registry study. The survival rate after 15 years was significantly lower in patients with relevant ITR compared to patients with no identifiable heart disease (25.8 ± 5 % *p* < 0.001) (14).

## Treatment Strategies for Tricuspid Regurgitation

Depending on the etiology and the morphology as well as the severity of TR and patient's risk factors individualized therapeutic regimes should be chosen. In the presence of risk factors for the development of TR echocardiographic assessments at least once per year should be considered (39).

There are two main points to consider when treating TR conservatively: Symptomatic treatment of patients, e.g., with diuretics and medication for heart failure and treatment of the underlying diseases (e.g., left-heart related pathologies)(39).

In the presence of relevant primary tricuspid regurgitation surgery is the treatment of choice (39, 40). In patients with relevant secondary tricuspid regurgitation, tricuspid surgery is recommended in combination with a surgical left heart treatment or when patients have already undergone prior cardiac surgery, yet suffer from symptomatic tricuspid regurgitation (39, 40). In patients with singular, but at least severe secondary tricuspid regurgitation, surgery represents a suitable treatment option despite high in-hospital mortality which is probably caused by too late admission with a remarkably end-organ damage (1). In general, surgical treatment of TR aims to reduce the annulus size and to restore the valve geometry. Thereby, annuloplasty with rigid rings seems to have a lower rate of recurrent TR than flexible devices or tricuspid valve reconstruction using the DeVega technique (1).

Taken together, the treatment of relevant tricuspid regurgitation remains challenging. Conservative therapy over longer periods usually results in refractoriness in diuretic treatment and surgery is unsuitable for patients with high operative risk (1, 14).

However, in recent years, transcatheter tricuspid valve interventions for TR have been evolved and show promising results so far. Similar to the mitral valve, edge-to-edge valve repair, direct annuloplasty and valve replacement are the most commonly used treatment strategies (41, 42).

Yet, device selection for transcatheter tricuspid valve intervention is still based on limited experiences. Generally, treatment of very severe TR is challenging for any reconstructive system. In the presence of large coaptation gaps annuloplasty should be favored whereas in the setting of leaflet tethering edge-to-edge could be more suitable (16).

## Conclusion and Clinical Perspective

Tricuspid regurgitation can develop as a result of multiple underlying disease processes, which in turn lead to different morphologic phenotypes of TR. Irrespective of its cause the presence of TR adversely affects clinical outcome. It is therefore high time that TR is not only recognized as an important treatment target but also as a prognostic factor. TR and also risk factors for the development of TR should therefore be assessed as a routine part in the work-up of most cardiologic disorders in order to better understand its natural course and thus to create appropriate individually tailored treatment strategies.

## Author Contributions

All authors listed have made a substantial, direct and intellectual contribution to the work, and approved it for publication.

## Conflict of Interest

The authors declare that the research was conducted in the absence of any commercial or financial relationships that could be construed as a potential conflict of interest.
